# Postcardiotomy VA-ECMO for refractory cardiogenic shock

**DOI:** 10.1186/s13019-017-0674-5

**Published:** 2017-12-19

**Authors:** Michael Charlesworth, Rajamiyer Venkateswaran, Julian M. Barker, Lee Feddy

**Affiliations:** 1 Department of Cardiothoracic Anaesthesia, University Hospital South Manchester, Southmoor Road, Manchester, M23 9LT UK; 2 Cardiac and Transplant Surgeon, University Hospital South Manchester, Manchester, UK; 3 Cardiothoracic Anaesthesia, Critical Care and ECMO, University Hospital South Manchester, Manchester, UK

**Keywords:** Postcardiotomy, VA-EMO, Systematic review, Cardiogenic shock

## Abstract

Postcardiotomy cardiogenic shock (PCCS) is a rare but catastrophic syndrome that can occur following separation from cardiopulmonary bypass or at any time during the immediate postoperative course. The management of PCCS varies between clinicians, institutions and countries. The available evidence to guide this practice is limited. In their systematic review and meta-analysis, Khorsandi and colleagues report a synthesis of case-series pertinent to the use of venoarterial extracorporeal membrane oxygenation (VA-ECMO) for PCCS. Whilst we acknowledge the potential survival benefit for carefully selected patients for what is ordinarily a condition with high mortality, we wish to comment on several aspects of the study in the context of its application to clinical practice.

In their systematic review and meta-analysis of venoarterial extracorporeal membrane oxygenation (VA-ECMO) for postcardiotomy cardiogenic shock (PCCS), Khorsandi and colleagues report a pooled survival to hospital discharge of 30.8% and suggest a number of adverse prognostic indicators [1]. Whilst we agree that postcardiotomy VA-ECMO for refractory cardiogenic shock does indeed provide a significant survival benefit, we wish to highlight several limitations so as to aid interpretation of this study and inform future analyses.

With regards the search strategy, it was unclear from which database(s) (Medline and/or PubMed) articles were retrieved and from when the search extends. A recent Cochrane review advised against the pooling of studies from prior to 2000 due to significant advances in technology, yet three of the included studies are from the 1990s [2]. The use of other databases, a Google™ search and searching the bibliography of included manuscripts could have ensured a more comprehensive strategy. With regards inclusion and exclusion criteria, all transplant and non-transplant patients receiving VA-ECMO for postcardiotomy cardiogenic shock were included. At our institution, it is generally more common for VA-ECMO to be employed following heart transplantation than it is following non-transplant cardiac surgery. Anecdotally, we also find that outcomes following planned VA-ECMO use following heart transplantation are better as compared to unplanned non-transplant postcardiotomy VA-ECMO. Given that transplant and non-transplant patients have very different baseline characteristics together with different pre, intra and postoperative courses, it may have been advisable to statistically treat transplant and non-transplant groups separately to further reduce data heterogeneity. The finding that all studies were observational in design and with many employing retrospective data collection, is shared by our systematic review of extracorporeal life support for postcardiotomy cardiogenic shock [3]. We also found considerable difficulty in retrieving all relevant articles due to variable definitions of PCCS and extracorporeal life support (ECLS) in the literature.

With regards outcome selection, the authors were limited by outcomes reported from individual studies. These outcomes varied as a function of survival time from mechanical support through to hospital discharge. As hospital discharge was commonly reported, this appears to have been pooled in the meta-analysis. Hospital discharge as a primary outcome has several limitations, as whilst it is commonly affected by non-clinical factors, it does not have a dependence on time and it tells us little about quality of life or other outcomes of interest to patients. With regards reported complication rates, retrospective data suffers from underreporting, and the true incidence is therefore difficult to determine. This is of great importance as a major barrier in the way of VA-ECMO commissioning in the UK is concern regarding complication rates. With regards the combination of outcomes in the meta-analysis, the authors are right to highlight the considerable data heterogeneity. This is not surprising given the design of the included studies. Whilst a critical appraisal of included studies together with survival data pooling is of great interest, it is uncertain what there is to be gained through more advanced statistical manipulation of these data.

Postcardiotomy VA-ECMO is an evolving science with a limited evidence base and with many barriers in the way of high-quality evidence generation [4]. This study poses ethical dilemmas regarding regional variations in decision-making, expertise and outcomes. As highlighted by Khorsandi et al., there are also significant ethical and logistical issues that preclude a randomised controlled trial. That said, this study demonstrates that VA-ECMO is far beyond experimental, as a significant number of patients are surviving that would not otherwise have done so. The decision to initiate postcardiotomy VA-ECMO is high-stakes due to the potential for poor outcomes from refractory PCCS without VA-ECMO on the one hand but with considerable resource expense on the other. In the UK, these costs are currently absorbed by individual hospitals. At our centre, the decision is taken following discussion and consensus between four appropriately experienced consultants. We have demonstrated our 30-day and 2-year survival rates to be 50% and as this is somewhat higher than the studies included by Khorsandi et al., we believe that we are currently getting these difficult decisions right through appropriate patient selection [5].

**Abbreviations**

ECLS, Extracorporeal life support; PCCS, Postcardiotomy cardiogenic shock; VA-ECMO, Venoarterial extracorporeal membrane oxygenation

**Acknowledgments**

None to declare

**Funding**

No funding

**Availability of data and materials**

Data sharing not applicable to this article as no datasets were generated or analysed during the current study.

**Authors’ contributions**

All authors contributed significantly to the drafting of this manuscript and all authorise the final version for consideration for publication.

**Ethics approval and consent to participate**

Not applicable

**Consent for publication**

Not applicable

**Competing interests**

The authors declare that they have no competing interests.

**Author details**

^1^Department of Cardiothoracic Anaesthesia, University Hospital South Manchester, Southmoor Road, Manchester M23 9LT, UK. ^2^Cardiac and Transplant Surgeon, University Hospital South Manchester, Manchester, UK. ^3^Cardiothoracic Anaesthesia, Critical Care and ECMO, University Hospital South Manchester, Manchester, UK.

**References**

1. Khorsandi M, Dougherty S, Bouamra O, Pai V, Curry P, Tsui S, Clark S, Westaby S, Al-Attar N, Zamvar V. Extra-corporeal membrane oxygenation for refractory cardiogenic shock after adult cardiac surgery: a systematic review and meta-analysis. J Cardiothorac Surg 2017;12:55. Available at: http://www.ncbi.nlm.nih.gov/pmc/articles/PMC5512816/.

2. Tramm R, Ilic D, Davies AR, Pellegrino VA, Romero L, Hodgson C. Extracorporeal membrane oxygenation for critically ill adults. Cochrane Database Syst Rev:CD010381.

3. Charlesworth M, Venkateswaran R, Barnard J, Barker J, Feddy L. Unplanned extracorporeal life support (ECLS) following cardiac surgery: a systematic review. Anaesthesia 2017;72(S4).

4. Charlesworth M, Venkateswaran R, Feddy L. When traditional research fails – the case for veno-arterial ECMO in postcardiotomy cardiogenic shock. Anaesthesia 2017; 72:1425.

5. Charlesworth M, Hernandez A, Feddy L, Barker J, Shaw S, Barnard J, Venkateswaran R. Post-cardiotomy extra corporeal life support (ECLS) for refractory cardiogenic shock: a 4-year retrospective case-note audit in South Manchester, UK. J Cardiothorac Vasc Anesth 2017;31:S83–4.

## Abbreviations

ECMO: extracorporeal membrane oxygenation
NLM: National library of medicine
UHSM: University Hospital of South Manchester
