# Supplementary material for: Postcardiotomy VA-ECMO for refractory cardiogenic shock
Source: J Cardiothorac Surg. 2017 Dec 19;12:116. doi: 10.1186/s13019-017-0674-5 (PMC5764222; doi:10.1186/s13019-017-0674-5)
Supplement: Related texts

# Reply: RE: Extra-corporeal membrane oxygenation for refractory cardiogenic shock after adult cardiac surgery: a systematic review and meta-analysis

**Authors:** Maziar Khorsandi, Scott Dougherty, Omar Bouamra, Vasudev Pai, Philip Curry, Steven Tsui, Stephen Clark, Stephen Westaby, Nawwar Al-Attar, Vipin Zamvar

**Affiliations:** 4 0000 0004 0590 2070 grid.413157.5 Department of Cardiac Surgery and Transplantation, Golden Jubilee National Hospital, Glasgow, UK; 5 0000 0000 9009 9462 grid.416266.1 Department of Cardiology, Ninewells hospital, Dundee, UK; 6 0000000121662407 grid.5379.8 Medical Statistics, Trauma, Audit & Research Network, University of Manchester, Salford Royal NHS foundation trust, Manchester, UK; 7 0000 0001 0571 5193 grid.411639.8 Department of Cardiovascular and Thoracic Surgery, Kasturba Medical College, Manipal university, Manipal, India; 8 0000 0004 0399 2308 grid.417155.3 Department of Cardiac Surgery and Transplantation, Papworth hospital, Cambridge, UK; 9 0000 0004 0641 3308 grid.415050.5 Department of Cardiac surgery and Transplantation, Freeman hospital, Newcastle, UK; 10 0000 0001 2306 7492 grid.8348.7 Department of Cardiac Surgery, Oxford Heart Center, John Radcliffe Hospital, Oxford, UK; 11 0000 0001 0709 1919 grid.418716.d Department of Cardiothoracic Surgery, Royal Infirmary of Edinburgh, Edinburgh, UK

**Keywords**

Postcardiotomy, ECMO, Cardiogenic shock, Systematic review

Dear Editor,

We would like to thank Charlesworth et al. for their letter. They have highlighted a few important points, which we would like to take the opportunity to reply to.

The PubMed search strategy has been clearly delineated under the methods subheading as three different searches (labelled 1–3). As far as we are concerned “MEDLINE” is the primary component of “PubMed” and a search in the latter should yield all the available articles on the topic from the “National Library of Medicine (NLM)”. MEDLINE provides 24 million references and PubMed contains 27 million (includes all of MEDLINE references) [6]. We have already stated in the methods section that the search was conducted with “no limit to the year of publication” in order to gain the maximum number of PubMed cited articles. We have also stated that we have performed the search in January of 2017.

In order to obtain the highest impact articles during this project we opted to exclusively look at PubMed/MEDLINE in order to avoid compiling excessive number of papers that would not change the message conveyed by the study. Hence we performed a cursory Google™ search only to ensure that we had caught the most high yield articles published up to that time. We also reviewed the bibliography of the obtained articles to look for any other studies, which were not captured in our original search.

The reason why we did not separate the postcardiotomy extracorporeal membrane oxygenation (ECMO) patient into transplant and non-transplant cohorts was the limited number of studies and the limited number of subjects within each study (table 1) [ 7]. We felt that in order for a meta-analysis to have the best chance at yielding any statistical significance these cohorts should be combined, albeit at the expense of greater heterogeneity. Furthermore, there is a great deal of variation in how each study has reported its outcomes and follow-up data and since there is no international consensus in reporting such data, this will continue to be a limitation in any systematic review reporting “post-intervention” outcomes.

We do regard the University Hospital of South Manchester (UHSM) very highly and would have certainly included their outcomes in our study had any outcome data been published at the time of the drafting of our manuscript. We believe that given the exceptionally high survival rate at the UHSM for postcardiotomy ECMO, it would be beneficial for the wider audience if any methods or protocols used at the UHSM to be shared.

Thank you.

Khorsandi et al.,
